# The Crusade against Tuberculosis

**Published:** 1898-07-23

**Authors:** 


### The Crusade Against Tuberculosis.

The days are long past when medicine was individual and medical questions lay exclusively between the doctor and his patient. Public medicine requires a widespread public knowledge of many of the secrets of disease, and, however much the more straight-laced and old-fashioned among us may deprecate the discussion of problems of pathology in the lay press, it is clear that so only can the outside multitude be led to take an interest in measures which are essential for their protection. When the *Practitioner* raised the banner of a new crusade and called upon mankind at large to join forces against that deadly enemy of the race—tuberculosis—its preaching would have fallen flat had it not been taken up and repeated on every side in daily papers, for it is only by the co-operation of the readers of these papers that any real progress can be made. We would point out, however, that while the "plan of campaign" to be adopted in this "crusade" concerns itself largely with the treatment of the developed disease, especially by fresh air in sanatoria for consumptives, the knowledge which has lately been gained as to the utility of open air in the treatment of the malady also teaches a much-needed lesson as to its prevention.

What is the meaning of the fact that consumption can be cured by life in the open air? What is the action of the enormous bulk of air which, when out of doors flows over and around the patient, but which he does not breathe? The meaning is that the air inside the house is foul and that the air outside is clean, that each consumptive patient while in the house tends to surround himself with a poisoned atmosphere infectious to himself as well as to his neighbours, and that when in the open what he expires at every breath is at once carried away and diluted with many thousands of times its own bulk of air which is pure and free from germs.

We may indeed well doubt how far it is in reality the fresh air which cures, and whether it is not rather the foul air which kills; and this brings us to our lesson, namely, that the more it is insisted that the true treatment of tuberculosis is by fresh air and sunshine, the more must we believe that its prevention is to be obtained by the same means.

Bacteriology is a science plentifully provided with pitfalls, and the difficulty which was at first experienced in cultivating the bacillus of tuberculosis gave a strong impetus to the idea that the infection from man to man must follow a fairly direct route. Hence at first much fear, and many exaggerated ideas as to the infectiousness of the disease. Then it was seen that in many cases it was in the form of dust from dried sputum that the infection was carried across, and a confidence perhaps almost as exaggerated arose in the efficacy of the spitting cup. "Let the sputum never dry," it was said, "and it can never become infective." But we have gradually got to know a good deal more about the habits of the bacillus of tuberculosis, and we now recognise that a by no means insignificant phase of its existence may be passed, not as a parasite of man, but as a clinger on to dirt, a saprophytic growth in condensed animal exhalations—in other words, that a much-lived-in house where the inhabitants are crowded, the air is scanty, and dirt and moisture are always present, becomes a home for the microbe in which it can not only rest but grow. Thus we come to see that, almost in the same way as plague, although it acts more slowly, tuberculosis is a house disease.

There is no doubt that there are houses in which every movement sets in motion clouds of tuberculous dust, which is ready to set up the disease if it is breathed by a person predisposed to the affection; but it is the unhygienic conditions of these houses that makes them so fit a breeding ground for these seeds of the disease. Let us then by all means advocate the removal of consumptives to sanatoria and their treatment by fresh air, and let us, when that cannot be done, advise the constant use of the spitting cup and the avoidance of all those habits by which the infection of tuberculosis tends to spread from person to person—let us, in fact, join heartily in the new crusade against tuberculosis; but let us not overlook the fact that before the germ origin of consumption was suspected people lived healthily in consumption hospitals, and this not by aid of any precautions against infection, but merely by virtue of fresh air and cleanliness, and that those are the true preventatives even now. Anyone when in a low state of health may become consumptive, but what keeps the malady alive among us is poverty and distress, dirt and overcrowding, darkness and lack of air. The cure of consumption may be attained by fresh air in the open, but its prevention is pure air in the house.

